# Transactional sex and risk for HIV infection in sub-Saharan Africa: a systematic review and meta-analysis

**DOI:** 10.7448/IAS.19.1.20992

**Published:** 2016-11-02

**Authors:** Joyce Wamoyi, Kirsten Stobeanau, Natalia Bobrova, Tanya Abramsky, Charlotte Watts

**Affiliations:** 1 Department of Sexual and Reproductive Health, National Institute for Medical Research, , Mwanza, Tanzania; 2 Department of Sociology, American University Institution, Washington, DC, USA; 3 International Centre of Research on Women, Washington, DC, USA; 4 Department of Global Health and Development, London School of Hygiene and Tropical Medicine, London, UK

**Keywords:** HIV, young women, adolescent, transactional sex, sub-Saharan Africa, sexual behaviour

## Abstract

**Introduction:**

Young women aged 15 to 24 years in sub-Saharan Africa continue to be disproportionately affected by HIV. A growing number of studies have suggested that the practice of transactional sex may in part explain women's heightened risk, but evidence on the association between transactional sex and HIV has not yet been synthesized. We set out to systematically review studies that assess the relationship between transactional sex and HIV among men and women in sub-Saharan Africa and to summarize the findings through a meta-analysis.

**Methods:**

The search strategy included 8 databases, hand searches in 10 journals, and searches across 17 websites and portals for organizations as informed by expert colleagues. A systematic review of cross-sectional and longitudinal studies was carried out for studies on women and men who engage in transactional sex published up through 2014. Random effects meta-analysis was used to further examine the relationship between transactional sex and prevalent HIV infection across a subset of studies with the same exposure period. Analyses were conducted separately for men and women.

**Results:**

Nineteen papers from 16 studies met our inclusion criteria. Of these 16 studies, 14 provided data on women and 10 on men. We find a significant, positive, unadjusted or adjusted association between transactional sex and HIV in 10 of 14 studies for women, one of which used a longitudinal design (relative risk (RR)=2.06, 95% confidence interval (CI): 1.22 –3.48). Out of 10 studies involving men, only 2 indicate a positive association between HIV and transactional sex in unadjusted or adjusted models. The meta-analysis confirmed general findings from the systematic review (unadjusted meta-analysis findings are significant for women (*n=*4; pooled odds ratio (OR)=1.54, 95% CI: 1.04–2.28; *I*^2^=42.5%, *p=*0.156), but not for men (*n=*4; pooled OR=1.47, 95% CI: 0.85–2.56; *I*^2^=50.8%, *p=*0.107).

**Conclusions:**

Transactional sex is associated with HIV among women, whereas findings for men were inconclusive. Given that only two studies used a longitudinal approach, there remains a need for better measurement of the practice of transactional sex and additional longitudinal studies to establish the causal pathways between transactional sex and HIV.

## Introduction

Although the HIV epidemic is generalized in sub-Saharan Africa, there is heterogeneity in where and among whom HIV infections occur, with certain localities and populations being consistently more vulnerable to infection than others [1,2]. For example, HIV prevalence among young women remains more than twice as high as in young men throughout sub-Saharan Africa [1]. Among those living with HIV, AIDS is now the leading cause of death among adolescents in Africa and the second most common cause of death among adolescents globally [2,3]. Given young women's continued disproportionate risk of HIV, prevention of HIV in adolescent girls and young women is a long-standing priority.

The disproportionately high HIV incidence in young women compared to young men has been attributed to social and economic aspects of gender inequality and to specific factors such as age disparate sexual relationships [4,5], poor negotiating power with respect to condom use [5–8] and intimate partner violence [9,10]. A growing body of literature speculates that transactional sex—defined here as *non-marital, noncommercial sexual relationships motivated by the implicit assumption that sex will be exchanged for material benefit or status* [11]—may play a role in young women's disproportionate risk and explain the feminization of the epidemic [2,12].

The term “transactional sex” emerged from efforts to differentiate Western connotations of “sex work” from the exchange practices embedded in many relationships in contexts outside of the West. Numerous in-depth studies conducted across the region confirm [11] first that transactional sex relationships are *non-commercial*; participants describe themselves as boyfriends and girlfriends, or lovers, not as clients and sex workers. Second, the exchange embedded in these relationships is *implicit*; it is not formally negotiated and may not immediately follow a sexual act. Finally, many of these relationships include shared emotional intimacy.

Despite growing evidence, there has not yet been an attempt to synthesize the strength of the association between transactional sex and HIV. We therefore conducted a systematic review and meta-analysis to determine the extent to which transactional sex is a risk factor for HIV in sub-Saharan Africa.

## Methods

### Search strategy

This systematic review of the relationship between transactional sex and HIV is a part of a larger comprehensive review assessing the state of knowledge on transactional sex in sub-Saharan Africa including its conceptualization, definition and measurement as well as its association with HIV and related risk behaviours [11]. The comprehensive search strategy was broad to accommodate these multiple aims and includes studies conducted through 2014. We included the following databases for peer-reviewed articles: PubMed, EMBASE, Global Health, POPline, Web of Science, ADOLEC, Scopus and Anthropology plus. Grey literature and national reports were searched through several websites: Google Scholar, UNAIDS, UNFPA, WHO, the World Bank, FHI, Population Council, PSI, USAID, CIDA, DFID, PEPFAR, OSI, HIV/AIDS Alliance, Guttmacher Institute, African Population and Health Research Centre (www.aphrc.org) and Population Reference Bureau. Experts’ suggestions were also sought to identify relevant peer-reviewed articles as well as grey literature papers and reports. Other sources included four surveys: Demographic and Health Survey (DHS), Integrated Biological and Behavioural Surveillance Survey, National Reproductive Health Survey and Second Generation HIV and STI Surveillance Survey. In addition, hand searches were conducted in the following journals: African Journal of Reproductive Health, African Health Sciences, African Journal of AIDS Research, East African Journal of Public Health, East African Medical Journal, African Affairs, Culture Health and Sexuality, Archives of Sexual Behavior, Gender and Development and Exchange on HIV/AIDS Sexuality and Gender.

The search terms for both peer-reviewed articles and grey literature were as follows: “transactional sex” or “survival sex” or “consumption sex” or “intergenerational sex” or “commodified sex” or “cross-generational sex” or “informal sex,” or “sex* exchange,” or “sex* trade” or “sugar daddy*,” or “globalization and sex*” or “modernity and sex*” and Africa. Both quantitative and qualitative studies were included. No time or types of article restrictions were applied to the search. The results from the searches were downloaded to the EndNote program where duplicates were eliminated. Within this broader search strategy, we developed specific criteria reviewed below that applied to the systematic review of the association between transactional sex and HIV.

### Criteria for study population inclusion and exclusion

The broader literature review, discussions with experts and our own contributions to the field informed our definition of transactional sex, as stated above. This definition served as the basis for the following inclusion criteria for the systematic review: transactional sex was examined in populations other than sex workers, bar workers, men who have sex with men or drug users; and transactional sex was measured as distinct from sex work. We restricted our review to studies conducted within sub-Saharan Africa.

### Types of studies and outcome measures included

In as much as possible, we made efforts to include studies that captured transactional sex, not sex work. We included only studies that operationalized transactional sex as “exchange of sex for money or gifts” or other specific forms of material support (e.g. food, clothes, alcohol and cosmetics). Where the operationalization of transactional sex was not clear from the text of the article, we contacted the corresponding author to determine whether the measurement used had been interpreted by the authors and participants as distinct from “sex work.”

Our central objective was to measure the association between transactional sex and HIV. We only included studies with a biological measure of HIV. Furthermore, studies had to provide or allow calculation of a measure of association (such as a χ^2^ test, or unadjusted or adjusted odds ratio (OD)). Both HIV prevalence and incidence measures were included from observational and intervention studies.

### Data extraction and management

Quantitative data extracted included characteristics of the study population, sample size, study location, measures and prevalence of transactional sex, and HIV prevalence or incidence. Furthermore, unadjusted and adjusted associations between HIV and transactional sex were extracted, and papers were subdivided by sex and age groups (young people only, e.g. 15–26 years; mixed age range or adults, e.g. 15–49 years). We sex-disaggregate our findings as men and women have different roles in transactional sex that may correspond to differences in HIV risk.

### Meta-analysis

The meta-analysis was conducted in STATA version 13.0. Random effects meta-analysis was used to examine the relationship between transactional sex and prevalent HIV infection across studies. The meta-analysis was performed separately for men and women. Only studies that included sex-disaggregated measures of *ever* having engaged in transactional sex were included in the analysis to reduce heterogeneity of exposure. We therefore excluded studies from the meta-analysis that measured transactional sex in the last 12 months, 4 weeks or with a recent sexual partner. The decision to focus the meta-analysis on prevalent HIV infection was taken as only one study identified in the systematic review measured incident HIV infection. Log odds ratios (and 95% confidence intervals (CIs)) of the association between transactional sex and HIV infection, where possible adjusted for age and sample design (otherwise crude), were analyzed using the *metan* command [13]. Where the age-adjusted OR was not reported in a paper, attempts were made to obtain it from the study authors. Heterogeneity of study results was assessed visually by examining forest plots and statistically using the χ^2^ test for heterogeneity and the *I* ^2^ statistic) [14,15]. Sensitivity analyses were performed, respectively, excluding a study with a population that differed from other included studies (15–19-year-olds attending reproductive health clinics in an urban slum, rather than a population-based sample) and studies in which the OR was not adjusted for age.

## Results

**Figure 1:**
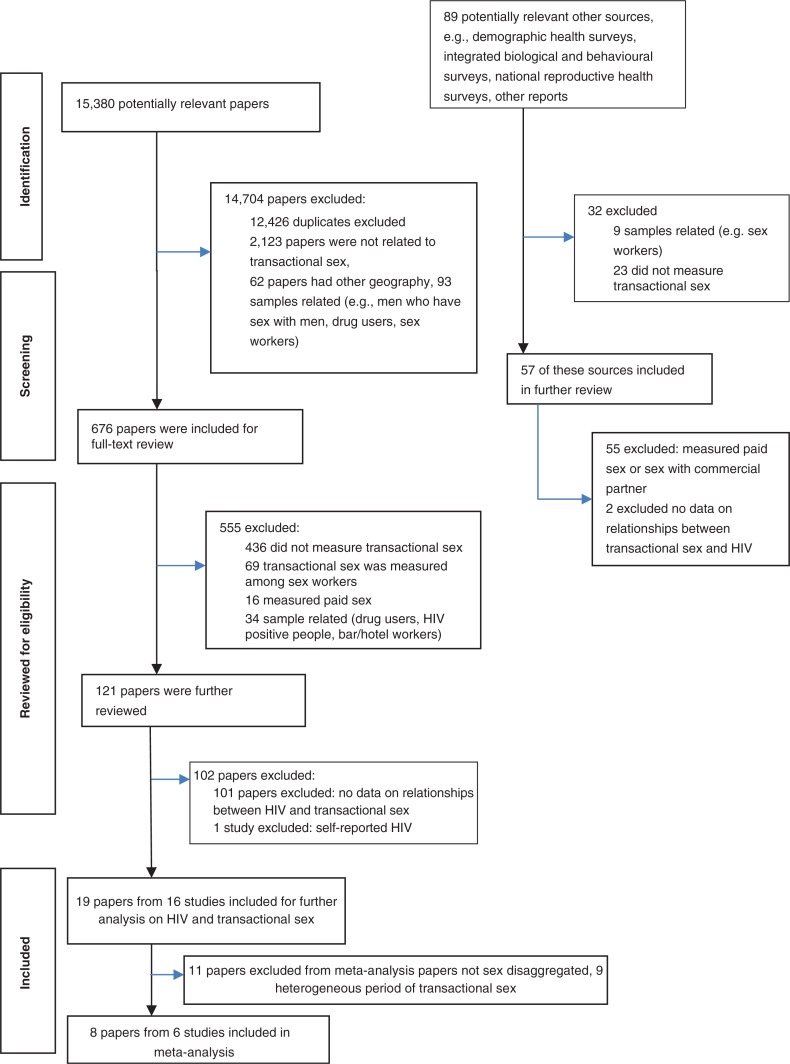
Flow chart of included studies.

The study selection process (studies on transactional sex and HIV) is summarized in the flow diagram in Figure 1. In brief, 15,380 records were identified for screening, of which 2954 were unique. We assessed 676 full-text articles for eligibility, from which 19 papers representing 16 studies met the inclusion criteria.

Studies that met eligibility criteria for inclusion in the systematic review are summarized in Table 1. The sample sizes ranged from 136 to 11,904. In total, 14 studies (15 papers) provide data on women, and 7 of these studies focused specifically on young women (age range 13–26 years). Ten studies provide data on men, four of which provide data exclusively on young men. Three studies provided measures of association that were not sex disaggregated [16–18]. Overall, 14 studies (17 papers) were cross-sectional or repeated cross-sectional and provide HIV prevalence measures, whereas two cohort studies provided HIV incidence measures. The majority of the studies set out to determine factors associated with HIV infection. In these studies, transactional sex was included as a predictor, but it was not the focus of the analysis. However, in three studies, the primary objective was to assess the role of transactional sex on HIV [19–21]. Of these three studies, one study used incident measures and was better able to assess the causal role of transactional sex in HIV risk [20]. The studies originate from a total of five different countries within southern and eastern Africa. The majority (8/16) were conducted in South Africa, and five were from Kenya.

**Table 1:** Details of studies included in a systematic review of the association between transactional sex and HIV for men and women in sub-Saharan Africa

Study	Setting	Objectives	Study design	Sample size	Participants characteristics	Age	Measure of transactional sex	Timing of exposure/recall period
**Young women**								
Gavin, 2006	Zimbabwe	To identify factors associated with HIV infection among adolescent females in Zimbabwe and appropriate prevention strategies for this vulnerable population	Cross-sectional	1807	Women recruited through household probability survey within nationally representative	15–19	Received money or goods in exchange for sex with *last partner*	Last partner
Rositch, 2012 (Included in the meta-analysis)	Kenya	To examine details of sexual behaviours and male partners that expose adolescent girls to HIV	Cross-sectional	761	adolescent girls seeking reproductive health care recruited from urban reproductive clinics	15–19	*Ever* had sex for money or favours	Ever
Ranganathan, 2016 (Included in the meta-analysis)	South Africa	To explore the relationship between self-reported transactional sex and HIV infection and to assess whether this relationship is mediated through certain HIV related risky behaviours	Cross-sectional	693	Sexually active rural young women from a large conditional cash transfer (CCT) trial in South Africa	13–20	Did you feel like you had to have sex with [Initials] because he gave you money or gifts or both	Ever
Jewkes, 2006a (Included in the meta-analysis)	South Africa	To describe factors associated with HIV serostatus in young, rural South African women and the relationship between intimate partner violence (IPV) and HIV	Baseline of RCT	1295	Sexually active rural women volunteers from 70 villages recruited to participate in randomized control trial (RCT) of an HIV behavioural intervention	15–26	*Ever* had a sexual relationship (or act) motivated by her expectation that he would provide her with food, cosmetics, clothes, transportation, items for	Ever
Jewkes, 2012	South Africa	To test hypotheses that transactional sex predicted incident HIV infections	Endline of RCT	1077		15–26	children or family, school fees, somewhere to sleep, alcohol or a “fun night out”, or cash	
**Young people (women and men)**								
Jewkes, 2006b (Included in the meta-analysis)	South Africa	To describe factors associated with HIV infection in men aged 15–26 years	Cross-sectional	1277	Sexually experienced Xhosa male volunteers from 70 villages participating in a cluster RCT of an HIV behavioural intervention	15–26	Ever had sex primarily motivated by material gain, where material gain was defined as provision of food, cosmetics, clothes, transportation, items for children or family, school fees, somewhere to sleep, or cash	Ever
Pettifor, 2005a (Included in the meta-analysis)	South Africa	To determine the prevalence of HIV infection, HIV risk factors, and exposure to national HIV prevention programmes and to identify factors for HIV infection among South African youth	Cross-sectional	11,904	Men and women, nationally representative household survey	15–24	Both men and women asked: Have you *ever* had sex with someone so that they would give you material or any other kind of support such as money, presents, alcohol, food, clothes, better grades, transportation etc. in exchange?” Men were also asked separately: about having given a woman any of these things in exchange for sex	Ever
Pettifor, 2005b	South Africa	To determine whether South African youths living in communities that had either of the two youth HIV prevention interventions would have a lower prevalence of HIV and STIs and high risk sexual behaviours than communities without either interventions	Repeated cross-sectional	8735	Men and women, in 33 communities, participated in the Love life campaign	15–24	Ever engaged in transactional sex	Ever
Mattson, 2007 (Included in the meta-analysis)	Kenya	To investigate sexual practices and risk factors for prevalent HIV infection among young men in Kisumu, Kenya	Cross-sectional	1337	Urban men, uncircumcised and had experienced sex within the last 12 months, recruited within the context of an RCT	18–24	Ever had sex with a women for money or gifts	Ever
**Mixed age studies among women, men, and women and men**								
Dunkle, 2004	South Africa	To estimate the prevalence of transactional sex among women attending antenatal clinics; to identify demographic and social variables associated with transactional sex with ‘roll-ons’; and to determine the association between transactional sex and HIV	Cross-sectional	1395	Women presenting for antenatal care at four health centres in Soweto, South Africa, who accepted routine antenatal HIV testing	16–44	*Ever* become involved with *a roll-on* because he provided you with or you expected that he would provide you with any of a list of commodities: food; cosmetics; clothes; transportation, school fees; somewhere to sleep, or cash	Ever
Dunkle, 2004b		To understand associations between HIV, gender-based violence and gender-based inequality in intimate partnerships, including transactional sex relationships with any non-primary partner		1366			*Ever* had sex with a non-primary partner (same operationalization of transactional sex as above)	
Serwadda, 1992 (Included in the meta-analysis)	Uganda	To examine the factors for HIV-1 infection	Cross-sectional	1292	Conducted in 21 randomly selected community clusters with rural Men and women	13 +	Exchanging sex for gifts or money	Likely either last 60 months or ever
Shaffer, 2010	Kenya	To report 36-month HIV-1 incidence rates and demographic and psychosocial risks from the Kericho cohort in rural Kenya's southern Rift Valley Province.	Prospective cohort	2400	HIV-negative rural men and women (not sex-disaggregated) in Kenya's southern Rift Valley Province	18–55	Providing sex for goods, Providing food for sex	Not stated, but may be only 6 months
Hunter, 1994	Kenya	To study risk factors for HIV	Cross-sectional	4404	Women attending 2 family planning urban clinics	15–49	Sex for gifts or money	Not stated in the article
Lohrmann, 2012	South Africa	To investigated the HIV prevalence and risk factors among urban homeless individuals in Johannesburg.	Cross-sectional	136	Adults (95% male) from a Johannesburg inner-city homeless clinic	Mean-32	Having sexual intercourse last 12 months	Last 12 months
Mmbaga, 2007	Tanzania	To investigate the magnitude of HIV-1 infection and identify HIV-1 risk factors that may help to develop preventive strategies in rural Kilimanjaro, Tanzania	Cross-sectional	1528	Individuals living in a rural village	15–44	Exchanging money/goods during last sex	Last sex
Nyaundi, 2011	South Africa	To determine the HIV prevalence and the factors associated with HIV infection in older South African women living in Soweto, Johannesburg.	Cross-sectional	449	Urban convenience sample of women who accepted to be tested for HIV, recruited from various venues in Soweto (a large urban African setting) in Johannesburg, South Africa	45+	Having had sex with a partner mostly motivated by material gain (e.g. food, clothes, cash, status, etc.) adapted from Dunkle, 2004	Ever
Chopra, 2009	South Africa	To collect HIV data from high-risk men who have multiple, younger, female sex partners in a peri-urban township in South Africa	Cross-sectional	421	High-risk peri-urban township men who have multiple, younger sex partners. Recruited through respondent-driven sampling	Mean-28	Giving any material goods to main partner/casual partner/1 time partner during recent sexual encounter	Recent sexual encounter
Kwena, 2010	Kenya	To assess prevalence and risk factors for sexually transmitted infections (STIs) among fishermen along Lake Victoria, Kenya	Cross-sectional	250	Fishermen recruited from beaches in Kisumu District using proportional-to-size sampling based on the number of registered boats per beach	18–65	Exchanging gifts including fish for sex	Among last three partners

Among the 11 papers from South Africa, five provide data from two data sources. Three of these papers report on findings from the Stepping Stones Trial in rural South Africa, and two report baseline findings for each sex [10,22]; and the final reports endline findings for women [20]; another two papers report findings from the same study of pregnant women in antenatal clinics in urban, South Africa [9,19]. We did not include more than one study from the same data source in our sex-disaggregated meta-analyses.

Most of the studies were observational, apart from four HIV behavioural intervention-based studies [10,20–24]. Nine studies draw from general population groups, whereas seven were conducted with specific populations: three studies of women attending reproductive health clinics in urban settings [9,19,25,26]; one study of adults in an urban homeless clinic [16]; one urban convenience sample [27]; one study of men with multiple young partners from a peri-urban township [28]; one of fishermen [29]; and one study of urban, uncircumcised sexually experienced men [23].

Five studies conducted among women [10,17,20,21,26] and four among men [17,22–24] met the inclusion criteria for the meta-analyses.

### Measurement of transactional sex

The measurement of transactional sex varied across the studies (see Table 1). Six of the studies drew from a more nuanced definition of transactional sex (sex *motivated by* material gain/gifts/money) that better distinguishes the practice from sex work [9,10,19–22,24,27,28]. Seven studies used a conventional measurement approach, asking about “sex in exchange for gifts or money” [18,23,25,29–32], and another two studies did not clearly state their measurement approach, but described transactional sex as distinct from sex work in the text of the article [16,17]. We included one study that measured transactional sex as “ever had sex for money” [26]. We included this study despite it poorly distinguishing transactional sex from sex work because it provided a measure of association among adolescent girls in a context outside of South Africa. However, given our concern about whether this measure adequately distinguished transactional sex from sex work, we run meta-analyses with and without this study [26].

The exposure period also varied across studies. In eight studies, respondents indicated whether they had “ever” practiced transactional sex. In six studies, the exposure period varied (e.g. transactional sex with “last partner” or “in the last 12 months”), and in two studies, the exposure period was not clearly stated.

### Associations between transactional sex and HIV

Tables 2 and 3 present prevalence or incidence statistics and measures of association between transactional sex and HIV. In the majority of cases, studies compare HIV rates between those who reported having practiced transactional sex with those who did not report transactional sex. However, a minority of studies present a comparison of transactional sex prevalence between respondents who are HIV positive compared to those who are HIV negative. It is important to note that three studies provide measures of association that are not sex disaggregated [16–18]. These studies appear in Tables 2 and 3.

**Table 2:** Measures of association between transactional sex and HIV among women in a systematic review of studies from sub-Saharan Africa

Study	Transactional sex prevalence	HIV prevalence	Descriptive measure of association between transactional sex and HIV		*p*	Unadjusted OR (95% CI)	AOR	Factors adjusted for
**Younger women (age 13–26)**								
Gavin, 2006	–	10.6%	Among HIV+: 37.9% report transactional sex	Among HIV−: 31.2% report transactional sex	0.58	–	–	–
Jewkes, 2006a	–	12.4%	Among HIV+: 12.7 report transactional sex	Among HIV-: 8.7 report transactional sex			1.09 (0.73–1.61)	Age (provided by author)
Jewkes, 2012	8.7%	6.2%	Transactional sex once off partner: 2.4% HIV IR Transactional sex main partner: 19.7% HIV IR Transactional sex ongoing casual partner: 12.6% HIV IR	Transactional sex once off partner 0.4% no HIV IR Transactional sex main partner 12.5% no HIV IR Transactional sex ongoing casual partner: 4.6% no HIV IR	0.046 – one-off partner 0.111 – main partner 0.007 – ongoing, casual partner	NR	One-off partner: IRR – 3.29 (1.02–10.55) Main partner: IRR – 1.44 (0.92–2.24) Casual partner: IRR – 2.06 (1.22–3.48)	Age, HSV-2, relationship power, condom use, IPV exposure, treatment, stratum, person years of exposure
Rositch, 2012	3%	7%	–	–	≤0.001	5.6 (2.2–14.1)	1.8 (0.5, 7.2)	Years of education, currently earn money, health clinic, years since sexual debut, number of partners last year, ever given birth, ever had non-consensual sex, ever exchanged sex for money, knowledge of HIV partner status
Ranganathan 2016	14%	5.8% of sexually active	Yes transactional sex: 10.5% (*n*=10) HIV+	No transactional sex: 5.1% (*n*=30) HIV+	0.05	2.2 (1.04–4.7)	2.4 (1.0–5.3)	Age of young woman, having a boyfriend, socio-economic status, type of primary caregiver, number of household members, age of first sex, orphan and work done for money
Pettifor, 2005a	2.1%	15.5%	Yes transactional sex −26.3%	No transactional sex −20.9%	–	1.3 (0.6–2.9)	–	
Pettifor, 2005b	15–19, 2.4% 20–24, 2.9%	20%	NR	NR	0.02	NR	1.86 (1.10–3.12) (Statistic is not sex disaggregated, reported the same AOR for both women and men)	Age, household wealth, education, study arm, sex, lifetime number of sexual partners, condom use with last partner, 10+ year older sexual partner, frequency of sex in last month, STIs
**Mixed age group women**								
Dunkle, 2004	21% ever	n.a.	NR	NR	NR	NR	1.54 (1.07–2.21)	Time from first coitus and lifetime number of male partners
Dunkle, 2004b	Not shown	33.5%	Yes transactional sex −44.8%	NR	NR	1·85 (1·42–2·41)	2.03 (1.10–3.77) <5 partners 1.69 (1.21–2.37) ≥5 partners	IPV, gender power difference, alcohol or drug problem
Serwadda, 1992	6.9%	24%	Yes transactional sex: 47.9% HIV+	No transactional sex: 22.2% HIV+	–	2.2 (1.2–3.8)	NS	Age, education, residence, occupation, partners, history of STD
Shaffer, 2010	4.3% provide food for sex 11% provide sex for goods	1.01, 36 month, IR (0.64–1.51)^a^	–	–	0.134 <0.001	Provide food for sex (men): HR – 1.64 (0.86–3.14) Provide sex for goods (women): HR – 3.30 (1.79–6.09)	Provide food for sex (men): HR – 1.40 (0.69–2.88) Provide sex for goods (women): HR – 2.99 (1.56–5.70)	Age (years), sex, education, and tribe
Hunter, 1994	Not shown	4.9%	Yes transactional sex: 5.8% HIV+	No transactional sex: 4.9% HIV +	NR	1.2 (0.5–2.7)	0.7(0.3–1.6)	Age, education, marital status, pregnancies, age at first sex, abortions, lifetime sex partners, sex partners in past year, sex during menstruation, circumcised partner, injection in past 6 months, transfusion in past 6 years, syphilis, trichomoniasis, gonorrhoea history, gonorrhoea culture
Mmbaga, 2007	8.2%	8.0 (age adjusted)	Yes transactional sex: 13.7% HIV+	No transactional sex: 8.9% HIV+	NR	NR	1.9 (0.8–4.2) among women	Adjusted for age, marital status, education level and religion
Nyaundi, 2011	30.4%	11.6%	Yes transactional sex: 20% HIV+	No transactional sex: 8.2% HIV+	<0.01	2.78 (1.36–5.69)	2.44 (1.04–5.69)	Adjusted for other variables in the model (not stated)

NS=not significant; NR=not reported – included variable in the analysis did not report the result; n.a.=not applicable.

a This was a prospective cohort study and we report in Table 2 that the cumulative HIV incidence at 36 months for women is 1.01 (95% CI=0.64–1.51).

**Table 3:** Measures of the association between transactional sex and HIV among men from a systematic review of studies from sub-Saharan Africa

Study	Transactional sex%	HIV%	Descriptive measure of relationship between transactional sex and HIV		*p*	Unadjusted OR (95% CI)	AOR (95% CI)	Factors adjusted for
Pettifor, 2005a	3.5%	5.9%	Report yes transactional sex: 4.7% HIV +	Report no transactional sex: 6% HIV+		0.8 (0.3–1.9)	–	–
Pettifor, 2005b	15–19, 2.9% 20–24, 4.3%	14.4%			0.02	NR	1.86, (1.10–3.12) (statistic not sex disaggregated)	Age, electricity in household, education, study arm, sex, time since last relationship, no of lifetime sex partners, condom use, age of partner, frequency of sex in last month, positive for gonorrhoea, self-reported genital ulcers, participated in love life
Mattson, 2007	36%	5%	Report yes transactional sex: 8% HIV+	Report no transactional sex: 3% HIV+	*p<*0.01	2.4 (1.5–4.0)	2.2 (1.3–3.7)	A final model was built by adding demographic characteristics (e.g. age) and behavioural risk factors that were significant in bivariate analyses
Jewkes, 2006b	17.8%	2%	Report yes transactional sex: 11.5 HIV+	Report yes transactional sex: 18% HIV−		NS	0.87 (0.36–2.13)	Age (provided by author)
Serwadda, 1992	5.6%	15%	Report yes transactional sex: 27.3 HIV+	Report no transactional sex: 14.1 HIV+		2.3 (1.0–5.4)	NS	All even slightly significant socio-demographic (e.g. age and residence,) and risk behaviour variables (e.g. sex partners, history of STIs and male circumcision) from univariate model were included in multivariate model
Shaffer, 2010	15.2% provided food for sex 5% provided sex for goods	1.00, 36 month, IR (0.71–1.36)^a^			0.134 <0.001	Provide food for sex (men): HR – 1.64 (0.86–3.14) Provide sex for goods (women): HR – 3.30 (1.79–6.09)	Provide food for sex (men): HR – 1.40 (0.69–2.88) Provide sex for goods (women): HR – 2.99 (1.56–5.70)	Age (years), sex, education and tribe
Lohrmann, 2012	13%	23.5%	HIV+: 7% report yes transactional sex	HIV−: 11% report yes transactional sex		NS/NR	NI	
Mmbaga, 2007	13%	3.2% (age adjusted)	Report yes transactional sex: 4.0% HIV+	Report no transactional sex: 3.8% HIV+		NR	1.0 (0.3–3.6)	Adjusted for age, marital status, education level and religion
Chopra, 2009	46% main partner 82.8% casual partner 90.6% one off partner	12.3%	Main partner, HIV+: 31.7 report yes transactional sex Casual, HIV+: 85.9% report yes transactional sex One-off, HIV+: 95.1% report yes transactional sex	Main partner, HIV−: 50% report yes transactional sex Causal, HIV−: 81.5% report yes transactional sex One-off, HIV−: 90.1% report yes transactional sex		Main partner: 0.68 (0.36–1.29) Casual partner: 1.23 (0.52–2.93) One-off: 1.40 (0.40–4.90)	NC	
Kwena, 2010	65%	26%				0.64 (0.30–1.36)	NI	

NS=not significant; NR=not reported – included variable in the analysis did not report the result; NC=not calculated; NI=not included – did not include the variable in the analysis.

a This was a prospective cohort study and we report in Table 3 that the cumulative HIV incidence at 36 months for men is 1.00 (95% CI=0.71–1.36).

### Young women

Across the six studies (seven papers) conducted among young women (≤26 years), the prevalence of reported transactional sex ranged from 2.1 to 14% (Table 2). Four studies report a significant unadjusted OR or test of association (with reported *p*-value) between transactional sex and HIV. Four studies report results from multivariate analyses. Although there are some distinctions (see Table 2), most studies controlled for age – particularly important for valid estimation with very young women – some measure of socio-economic status, a series of related sexual behaviours (e.g. number of partners, condom use and age of sexual debut) and some also included relationship characteristics. Of these, one study with a highly significant unadjusted OR lost significance in the adjusted model [26]. The remaining three studies (including one study not disaggregated by sex) report a significant adjusted OR, indicating that those who had practiced transactional sex had nearly two to more than three times the risk of being HIV positive [10,17,20,21]. One of these studies, using a longitudinal design, reported an increase in HIV incidence resulting from transactional sex for two partner types: casual partners (incidence rate ratio (IRR)=2.06, 95% CI: 1.22–3.48) and “one-off” (one time only) partners (IRR=3.29, 95% CI: 1.02–10.55) [20].

### Women of mixed age groups

Among the six mixed age group studies, the prevalence of transactional sex ranged from 4.3 to 30.4% (Table 2). Four of the studies report a significant unadjusted OR [9,18,27,32]. In all of these studies, adjusted ORs were also reported. Of the studies that provided details about the multivariate analyses, all models were adjusted for age and socio-economic characteristics; some also controlled for sexual behaviours/outcomes and relationships characteristics. In one study, the association loses significance in the adjusted model, perhaps due to over-adjustment [32]. In total, three studies (represented in four papers) provide a significant adjusted OR for the association between transactional sex and HIV, one of which is longitudinal (hazard ratio=2.99, 95% CI: 1.56–5.70) [18]. These studies find that women of mixed age groups who report transactional sex were >1.5 times up to nearly 3 times more likely to be HIV infected.

**Figure 2:**
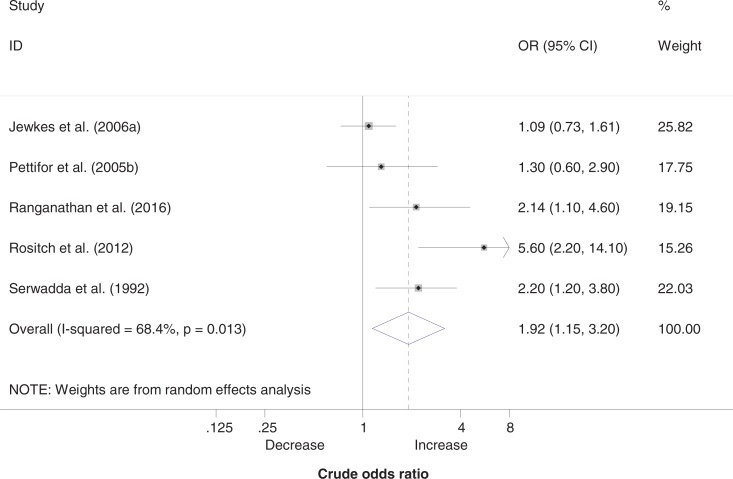
Association between transactional sex and HIV in women.

Figure 2 shows the results of the meta-analysis of the relationship between transactional sex and prevalent HIV infection among women of all age groups. All five ORs relating to women were >1, indicating a positive relationship between transactional sex and HIV, although the magnitude of point estimates ranged from 1.09 to 5.60. Three of the five ORs were statistically significant. The pooled OR, interpreted as the average association between transactional sex and HIV infection (assuming it may be different in different populations and study settings), was estimated at 1.92 (95% CI: 1.15–3.20). However, substantial heterogeneity was observed between studies (*I* ^2^=68%, *p=*0.013), potentially undermining the utility of a pooled estimate.

Exclusion of one study [26] in which the study population was not entirely comparable with the others led to a considerable reduction in heterogeneity between the studies (*I* ^2^=42.5%, *p=*0.156), making calculation of a pooled OR valid. This pooled OR confirmed a positive association between transactional sex and HIV infection (pooled OR=1.54, 95% CI: 1.04–2.28) among women.

Exclusion of studies in which the OR was not adjusted for age left us with only two studies: one study showed a large and statistically significant association (OR=2.14, 95% CI: 1.10–4.60) [21] and the other study showed no association (OR=1.09, 95% CI: 0.73–1.61) [10].

### Men

The reported transactional sex prevalence in studies among men (Table 3) ranged from 3.5% [24] to as high as 90.6% in a study of “high-risk” men with multiple younger one-off partners [28]. Out of 10 studies, only 3 studies indicate a positive association between HIV and transactional sex in unadjusted or adjusted models [23,32]. Two studies report significant findings in adjusted models: one study among urban, uncircumcised men in Kenya [23] and one study that is not sex disaggregated [17]. Furthermore, in three of these studies although the measure of association is not significant, the point estimate indicates a negative association between transactional sex and HIV [16,24,29].

**Figure 3:**
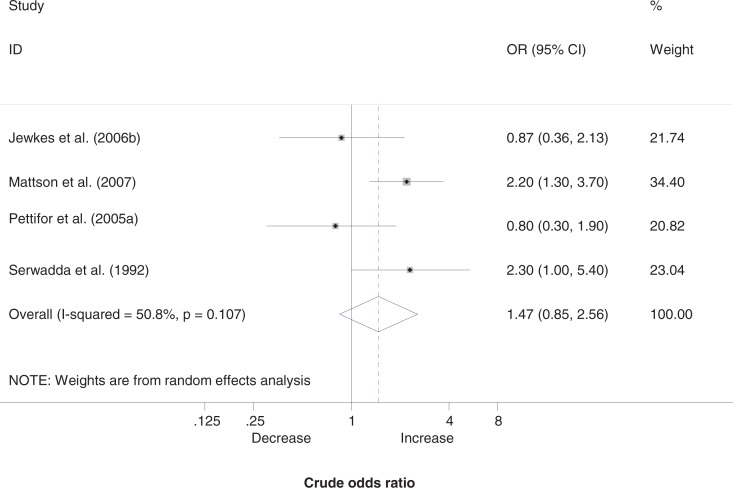
Association between transactional sex and HIV in men.

Figure 3 shows the results of the meta-analysis for men. Two of the four ORs pointed to a large and statistically significant positive relationship between transactional sex and HIV infection [23,32], whereas two indicated a weak (and statistically non-significant) inverse association [22,24]. The pooled OR was 1.47 (95% CI: 0.85–2.56), although moderate-to-substantial levels of heterogeneity between studies (*I* ^2^=50.8%, *p=*0.107), combined with inconsistency in the direction of association, make this estimate of “average” association potentially misleading.

Exclusion of studies in which the OR was not adjusted for age left us with only two studies: one study showed a large and statistically significant association (OR=2.20, 95% CI: 1.30–3.70) [23] and the other study showed a small and non-significant negative association (OR=0.87, 95% CI: 0.36–2.13) [22].

## Discussion

The results of this systematic review and meta-analysis of the association between transactional sex and HIV among men and women in sub-Saharan Africa indicate that transactional sex is a risk factor for HIV infection among women. Evidence from the systematic review, meta-analysis and both longitudinal studies, providing incident measures, all corroborate this assertion and suggest that women who practice transactional sex in sub-Saharan Africa are between 1.5 and nearly 2 times more likely to be infected with HIV. Our findings with respect to this association among men, however, are far less conclusive and indicate that transactional sex may not increase men's risk of HIV.

We found only one sex-disaggregated longitudinal analysis of the relationship between transactional sex and HIV, demonstrating the need for additional longitudinal studies that can rigorously examine the causal pathways between transactional sex and HIV. However, there is already some evidence regarding different mechanisms through which transactional sex might increase the risk of HIV for women. Several studies included in this review also assessed the association between transactional sex and other known HIV risk behaviours and outcomes. These studies have begun to uncover plausible pathways linking transactional sex with HIV [29,33,34]. At the individual and interpersonal level, transactional sex has been associated with alcohol use, history of having experienced intimate partner violence, multiple and concurrent partnerships, age-disparate sex and nonuse of condoms [9,10,19,20,22,29,34–38]. Because some of the adjusted ORs presented in this review adjust for such variables (i.e. factors potentially on the causal pathway between transactional sex and HIV), they are likely to be underestimates of the true association between transactional sex and HIV. Due to concern about the potential for over-adjusting, the meta-analysis used ORs that had been adjusted only for age (where possible). Finally, more work is needed to better understand whether and how transactional sex mediates the relationship between these risk behaviours and HIV. Models that assess such proximate behavioural determinants also need to better account for the structural drivers of HIV risk and transactional sex including social and economic aspects of gender inequality.

In assessing the operationalization of transactional sex in the literature, we found studies used a range of measures [11] and transactional sex was too often conflated with “sex work” or “prostitution” in meaning and measurement [39–41]. We acknowledge that transactional sex and sex work exist along a continuum; therefore, we should not expect to be able to clearly distinguish the practices in every case. However, conflating these practices is problematic as it confounds efforts to track and understand the role that transactional sex may play in HIV risk, and stymies effective intervention efforts [11]. To effectively capture the contribution of transactional sex to the HIV epidemic in sub-Saharan Africa where this practice is common, there is an urgent need to improve the measurement of this practice. An improved measure is particularly critical for large, repeat nationally representative surveys.

One consequence of the current tendency to conflate sex work and transactional sex is that we were unable to include several studies and data sources that could have contributed to our understanding of this relationship (69 studies and 55 DHSs were dropped due to weak measurement of transactional sex). Although we had intended to focus on the relationship between transactional sex and HIV among young people, limited evidence within this study population necessitated that we expand our search to all age groups. Age-disaggregated results suggest that transactional sex may be a significant risk factor for younger women, as well as women across their reproductive lifespan. However, effect sizes were generally larger in younger women than in older women, possibly due to their having less power in their relationships, poorer condom negotiation skills and more frequent engagement in risky behaviours including age-disparate sex.

The poor measurement of transactional sex may in part explain our inconsistent findings for men. Ethnographic studies, however, have provided a consistent depiction of the gendered relationship expectations that structure transactional sex across the region. Men are almost always expected to be the providers of material and financial support in transactional sex exchange [11,42–52]. Although it is important to mention that men are occasionally the recipients of goods or both recipients as well as providers [53,54], questions that aim to assess men's participation in transactional sex should prioritize their role as providers of goods in exchange for sex. Yet, our review found that in 4 of 10 studies examining transactional sex and HIV among men, the measurement questions for men were identical to those asked of women [17,18,24,29], presuming that they, too, were exchanging sex for goods. Men should be asked questions about both their participation as providers and their participation as recipients to reflect the gendered nature of the practice and to strengthen our understanding of the association between transactional sex and HIV. Our largely negative findings may also reflect that men are not as vulnerable within transactional sex relationships as women, an interpretation consistent with a wide literature documenting the unequal gender dynamics inherent in exchange-based relationships [20,55].

### Strengths and limitations of this review

To our knowledge, this study is the first systematic review to quantitatively assess the association between transactional sex and HIV in sub-Saharan Africa. This review points to many limitations in the existing epidemiological data. First, the evidence is overwhelmingly from South Africa, an area with high HIV prevalence, rendering the generalizability of these findings to other part of the sub-Saharan Africa region less clear. Second, most of the studies used a cross-sectional design (only two were longitudinal) [18,20]; therefore, we cannot assess temporality of the association. Third, heterogeneity in study population (health clinic attendees, rural population-based sample, intervention recipients), sample size (136–11,904) and sample frame (convenience sample, respondent-driven sample, random sample) among studies made it challenging to pool point estimates, and indicate caution must be made in generalizing these findings to young people and unrelated populations. Fourth, measures of transactional sex and control variables differed, making cross-study comparisons more challenging, and not all studies with young people controlled for age or years since sexual debut. Finally, three of these studies failed to examine this association in sex-disaggregated models, rendering the interpretation of the results more difficult. Despite these limitations, this review provides a strong case for the association between transactional sex and HIV among women in southern and eastern Africa, and demands that we continue to work toward better understanding how transactional sex contributes to women's risk of HIV.

## Conclusions

Overall, this review provides a needed summary of the state of the epidemiological evidence examining the association between transactional sex and HIV in sub-Saharan Africa. Our review confirms the epidemiological importance of transactional sex for women's risk of HIV in sub-Saharan Africa. This review also demonstrates important gaps that must be filled. We need additional longitudinal studies that use robust measures of transactional sex to further the understanding of the pathways through which transactional sex increases young women's risk of HIV. Such studies must account for social and structural drivers as well as contribute to our understanding of these dynamics across the many understudied settings in the region.
